# Anti-*Salmonella* Activity Modulation of Mastoparan V1—A Wasp Venom Toxin—Using Protease Inhibitors, and Its Efficient Production via an *Escherichia coli* Secretion System

**DOI:** 10.3390/toxins9100321

**Published:** 2017-10-13

**Authors:** Yeon Jo Ha, Sam Woong Kim, Chae Won Lee, Chang-Hwan Bae, Joo-Hong Yeo, Il-Suk Kim, Sang Wan Gal, Jin Hur, Ho-Kyoung Jung, Min-Ju Kim, Woo Young Bang

**Affiliations:** 1 Swine Science and Technology Center, Gyeongnam National University of Science and Technology, Gyeongnam 52725, Korea; chakfhd@daum.net (Y.J.H.); swkim@gntech.ac.kr (S.W.K.); iskim@gntech.ac.kr (I.-S.K.); sangal@gntech.ac.kr (S.W.G.); 2 National Institute of Biological Resources (NIBR), Environmental Research Complex, Incheon 22689, Korea; chaewon326@korea.kr (C.W.L.); bae0072@korea.kr (C.-H.B.); y1208@korea.kr (J.-H.Y.); 3 Veterinary Public Health, College of Veterinary Medicine and Bio-Safety Research Institute, Chonbuk National University, Iksan 54596, Korea; hurjin@jbnu.ac.kr; 4 Komipharm International Co. Ltd., Gyeonggi 15094, Korea; pignvet@gmail.com; 5 Department of Alternative Medicine, Kyonggi University, Gyeonggi 16227, Korea; only1only1@naver.com

**Keywords:** AMP, bacterial secretion system, inoculum effect, mastoparan, MP-V1, protease inhibitor, *Salmonella*, wasp venom toxin

## Abstract

A previous study highlighted that mastoparan V1 (MP-V1), a mastoparan from the venom of the social wasp *Vespula vulgaris*, is a potent antimicrobial peptide against *Salmonella* infection, which causes enteric diseases. However, there exist some limits for its practical application due to the loss of its activity in an increased bacterial density and the difficulty of its efficient production. In this study, we first modulated successfully the antimicrobial activity of synthetic MP-V1 against an increased *Salmonella* population using protease inhibitors, and developed an *Escherichia coli* secretion system efficiently producing active MP-V1. The protease inhibitors used, except pepstatin A, significantly increased the antimicrobial activity of the synthetic MP-V1 at minimum inhibitory concentrations (determined against 10^6^ cfu/mL of population) against an increased population (10^8^ cfu/mL) of three different *Salmonella* serotypes, Gallinarum, Typhimurium and Enteritidis. Meanwhile, the *E. coli* strain harboring *OmpA SS::MP-V1* was identified to successfully secrete active MP-V1 into cell-free supernatant, whose antimicrobial activity disappeared in the increased population (10^8^ cfu/mL) of *Salmonella* Typhimurium recovered by adding a protease inhibitor cocktail. Therefore, it has been concluded that our challenge using the *E. coli* secretion system with the protease inhibitors is an attractive strategy for practical application of peptide toxins, such as MP-V1.

## 1. Introduction

*Salmonella* infection is a major public health concern causing a primary enteric pathogenic disease in both humans and animals [1,2,3]. For example, *Salmonella* serotypes, such as Typhi and Gallinarum, cause typhoid fever—an acute illness—in human and domestic poultry species, respectively, and nontyphoidal *Salmonella* serotypes, including Typhimurium and Enteritidis, are the most common cause of foodborne infections [4,5,6]. Therefore, various antibiotics have been widely used for prevention and treatment of the infection, but this has caused the emergence and rapid dissemination of antibiotic-resistant bacteria, leading to serious problems with global human deaths due to antibiotic-resistant infections [7]. For this reason, recent studies have highlighted the discovery of novel and potent antimicrobial agents, including alterative drugs based on antimicrobial peptides (AMPs) [3,8,9,10,11,12].

The most promising candidates for AMPs have been discovered extensively in the venom of animals such as scorpions, snakes, spiders, ants, wasps, bees, centipedes, and so on. [13]. For example, peptide toxins, such as androtonin, parbutoporin, opistoporins, TstH and vpAmp 1.0, from scorpion venom, showed potent antimicrobial activity against Gram-positive and Gram-negative bacteria or fungi [14,15,16,17]. Cardiotoxin and crotamine from snake venom also exhibited potent antibacterial or antifungal activity [18,19]. Particularly, wasp and spider venoms offer a vast source of AMPs due to their diversity around the globe, with more than 20,000 and almost 40,000 species, respectively; mastoparans are representative AMPs from wasp venoms, and toxins including lycotoxins, lactarcins, oxyopinins and lycosin-II were identified in spider venoms [13].

Even though the toxins originated from venoms have been identified extensively as potent AMPs, there exist some limits for their practical application. For example, the AMPs can be subject to proteolytic degradation by proteases produced from an increased bacterial population [20,21,22], which may limit their pharmaceutical, nutraceutical and cosmeceutical uses. In addition, there are limits for their large-scale production because the chemical synthesis of large amounts of AMPs is unavailable in low unit cost and the over-collection of crude venom extracts for purification of AMPs can cause ecosystem destruction [23,24]. To the best of our knowledge, this report is the first that addresses these issues.

Recently, we reported that the mastoparan V1 (MP-V1), a de novo type of mastoparan from venom of the social wasp *Vespula vulgaris*, has superior anti-*Salmonella* activity compared with other typical mastoparans [25]. In this study, we also successfully modulated its antimicrobial activity against an increased *Salmonella* population through the use of protease inhibitors to overcome the proteolysis. In addition, we first made a cell-free supernatant including the MP-V1 with potent antimicrobial activity using an *Escherichia coli* secretion system. Therefore, our study supplies important information to set new strategies to modulate the antimicrobial activity of venom toxins and to produce them effectively for their practical application.

## 2. Results

### 2.1. Antimicrobial Activity of Synthetic MP-V1 against the Three Salmonella Serotypes

**Figure 1:**
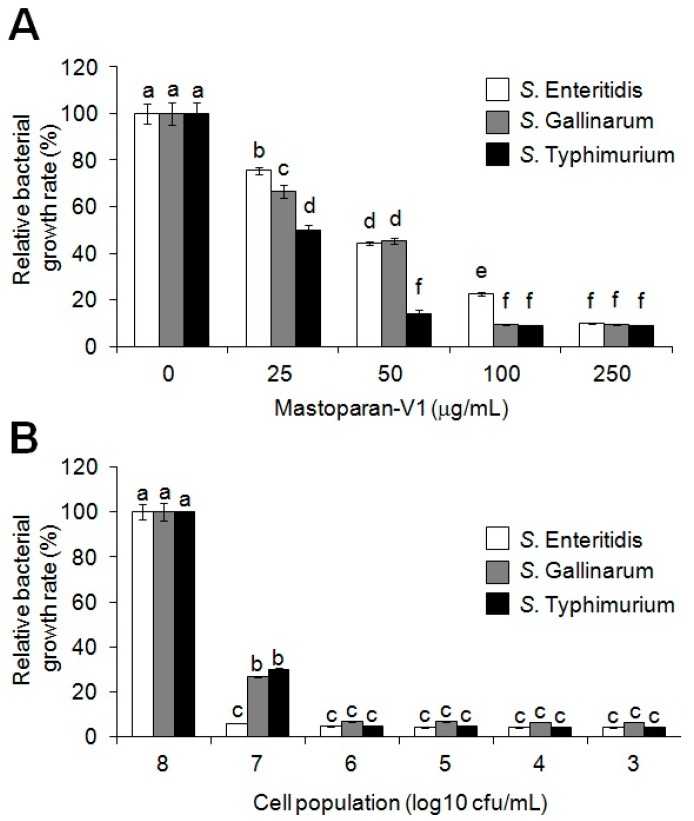
Antimicrobial activity of mastoparan V1 (MP-V1) against three *Salmonella* serotypes. (**A**) Determination of minimum inhibitory concentration (MIC) at against the three *Salmonella* serotypes, *Salmonella* Enteritidis, *Salmonella* Gallinarum and *Salmonella* Typhimurium, shown in Table 1. MIC of the synthetic MP-V1 was determined by using 25 to 250 μg/mL doses against 10^6^ CFU/mL of the *Salmonella* serotypes. (**B**) Examination of antimicrobial activity of the MP-V1 according to the *Salmonella* population density. Antimicrobial activity of the MP-V1 was examined with the MICs, determined by 10^6^ cfu/mL, against 10^3^ to 10^8^ cfu/mL of *Salmonella* population. Data are means ± standard error (SE) (*n* = 3). Different letters indicate significant differences by the one-way ANOVA/Duncan (*p* < 0.05).

Antimicrobial activity of the synthetic MP-V1 used in the previous study [25] was examined with 25 to 250 μg/mL concentrations against 10^6^ cfu/mL of three different *Salmonella* serotypes, Gallinarum—the typhoidal serotype—and Typhimurium and Enteritidis, the nontyphoidal serotypes (Table 1), as shown in Figure 1A. The minimum inhibitory concentrations (MICs) were determined as 106.95, 56.86 and 123 μg/mL against the three serotypes, Gallinarum, Typhimurium and Enteritidis, respectively. Subsequently, antimicrobial activity of the MP-V1 was examined with the MICs against 10^3^ to 10^8^ cfu/mL of *Salmonella* population (Figure 1B). MP-V1 at the MICs, determined by 10^6^ cfu/mL, significantly inhibited the bacterial growth against the 10^3^ to 10^7^ cfu/mL of the three different serotypes (Figure 1B). However, it showed no antimicrobial activities against the 10^8^ cfu/mL of population in all three serotypes (Figure 1B). Further challenges to recover its antimicrobial activities against the 10^8^ cfu/mL of population were performed as reported in the following section.

### 2.2. Anti-Salmonella Activity Modulation of the Synthetic MP-V1 Using Various Protease Inhibitors

**Figure 2:**
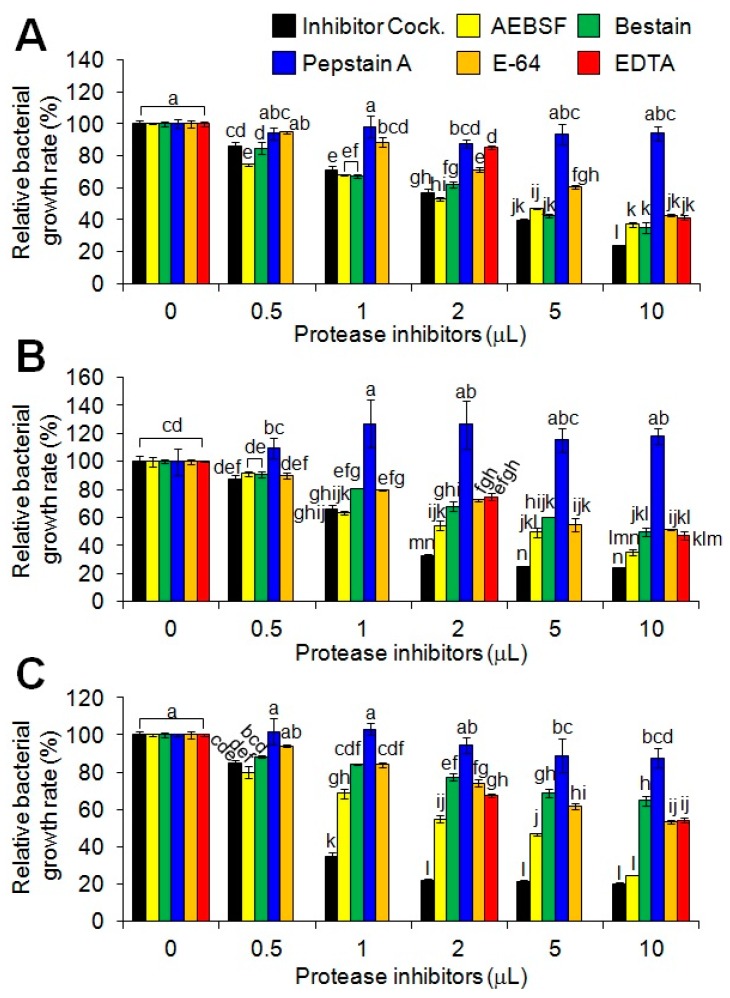
Effect of protease inhibitors on antimicrobial activities of MP-V1 in the increased *Salmonella* population density. The effect of a protease inhibitor cocktail (Sigma-Aldrich, Milwaukee, WI, USA) and its components (23 mM 4-(2-aminoethyl)benzenesulfonyl fluoride (AEBSF), 2 mM bestatin, 0.3 mM pepstatin A, 0.3 mM E-64 and 100 mM EDTA) on antimicrobial activities was examined using 0.5 to 10 μL doses against 10^8^ cfu/mL of *Salmonella* Enteritidis (**A**); *Salmonella* Gallinarum (**B**); and *Salmonella* Typhimurium (**C**). The MP-V1 was used at the MICs determined in 10^6^ cfu/mL. Data are means ± SE (*n* = 3). Different letters indicate significant differences by the one-way ANOVA/Duncan (*p* < 0.05).

**Figure 3:**
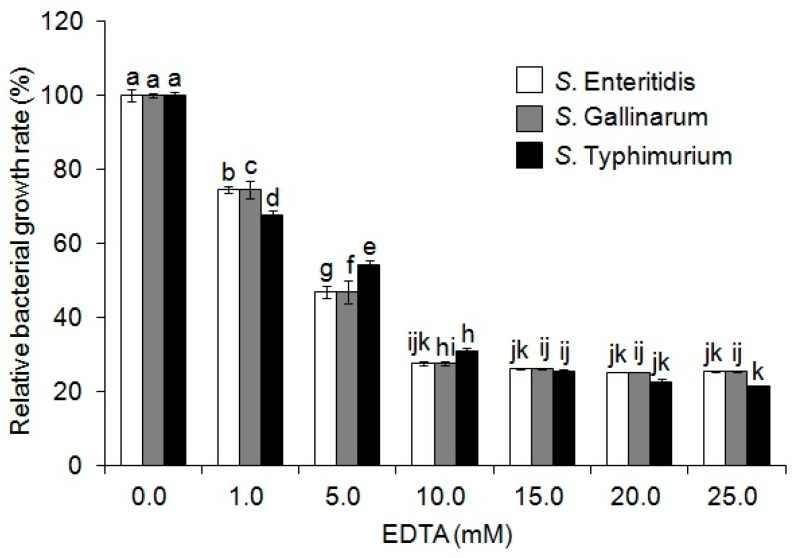
Effect of EDTA on antimicrobial activities of MP-V1 in the increased *Salmonella* population density. The effect of EDTA on antimicrobial activities was examined using 1 to 25 mM doses against 10^8^ cfu/mL of *Salmonella* Enteritidis, *Salmonella* Gallinarum and *Salmonella* Typhimurium. The MP-V1 was used at the MICs determined in 10^6^ cfu/mL. Data are means ± SE (*n* = 3). Different letters indicate significant differences by the one-way ANOVA/Duncan (*p* < 0.05).

Previous studies have highlighted that bacteria have an intrinsic AMP resistance mechanism through proteolysis using their proteases [21,22]. Thus, we here investigated the effect of a protease inhibitor cocktail (Sigma-Aldrich, Milwaukee, WI, USA) on antimicrobial activities of MP-V1 with MICs against 10^8^ cfu/mL of the three different *Salmonella* serotypes. The protease inhibitor cocktail exhibited a dose-dependent effect on the increase of the antimicrobial activity against 10^8^ cfu/mL of the all three serotypes (Figure 2). Next, each of the inhibitors were independently assessed as to whether they also have an effect on the increase of antimicrobial activity because the protease inhibitor cocktail consists of various inhibitors, such as 23 mM 4-(2-aminoethyl)benzenesulfonyl fluoride (AEBSF), 2 mM bestatin, 0.3 mM pepstatin A, 0.3 mM E-64 and 100 mM ethylenediaminetetraacetic acid (EDTA) (Figure 2). Except for pepstatin A, all inhibitors used showed a significant dose-dependent effect on the increase of antimicrobial activity against 10^8^ cfu/mL of all three serotypes (Figure 2). Among them, AEBSF in particular exhibited the most superior effect on the increase of antimicrobial activity against all three serotypes (Figure 2). Furthermore, EDTA, whose unit price is the lowest among the inhibitors, was shown to effectively increase the antimicrobial activity against the increased population of all three serotypes (Figure 3). These results indicate that MP-V1 is subjected to the proteolysis by bacterial proteases in the increased *Salmonella* population, and thus, protease inhibitors can be used as effective tools to modulate its antimicrobial activity.

### 2.3. Construction of the E. coli Secretion System to Efficiently Produce Active MP-V1

**Figure 4:**
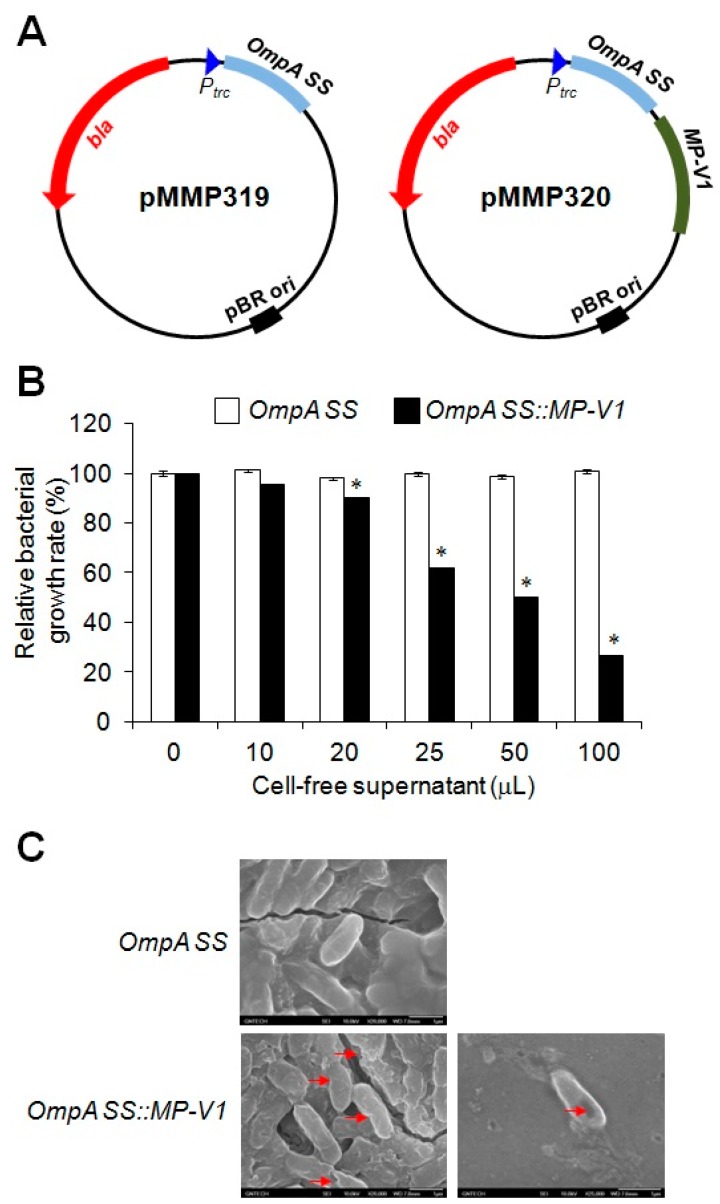
Examination of antimicrobial activity with the *OmpA SS::MP-V1* secretion system. (**A**) Construction of an *E. coli* secretion system for the production of MP-V1. The *MP-V1* sequence was designed to be directly fused with the *OmpA SS*, connecting to the translational start-site derived from the *Ptrc* promoter. The *OmpA SS*, fused directly to the one from the *Ptrc* promoter without the *MP-V1*, was designed to be used as a negative control. The designed artificial genes were cloned into the T-vector, finally resulting in the pMMP319 (left) and pMMP320 (right), respectively. (**B**) Comparison of antimicrobial activity of the *OmpA SS::MP-V1* secretion system with that of the *OmpA SS* one. Antimicrobial activity of the cell-free supernatants from the *OmpA SS::MP-V1* strain, an *E. coli* cell harboring *pMMP320*, and the *OmpA SS* strain, an *E. coli* cell harboring *pMMP319*, was examined with 10 to 100 μL doses against 10^6^ cfu/mL of *Salmonella* Typhimurium. Data are means ± SE (*n* = 3). Asterisks indicate significant effect of the *OmpA SS::MP-V1* strain as compared to the *OmpA SS* one by the two-way ANOVA/Duncan (*p* < 0.05). (**C**) Scanning-electron micrographs of *Salmonella* Typhimurium treated with the cell-free supernatants from the *OmpA SS::MP-V1* strain and the *OmpA SS* one. The red arrows indicate the pores forming into the *Salmonella* membrane.

The second aim for this study is to efficiently obtain the MP-V1 with antimicrobial activity at a low unit price for a practical application. Here, we constructed an *E. coli* secretion system to efficiently express active MP-V1 peptides, subsequently secreted into the cell-free supernatant by the Sec-dependent type II secretion system, consisting of Sec and GSPs (general secretory proteins) [26,27,28]. The OmpA signal sequence (OmpA SS) was used as a signal peptide for the secretion of MP-V1 through the type II secretion system. In short, the nucleotide sequence fused with the *OmpA SS* and the *MP-V1* sequence were prepared by an artificial gene synthesis, and finally were cloned as the pMMP320 plasmid (Figure 4A). 

To identify the secretion of MP-V1 into the cell-free supernatant, the cell-free supernatant from the *OmpA SS::MP-V1* strain, an *E. coli* cell harboring *pMMP320*, was subjected to the examination of antimicrobial activity, which was performed with 10 to 100 μL doses against 10^6^ cfu/mL of *Salmonella* Typhimurium (Figure 4B). The *OmpA SS::MP-V1* strain dose-dependently inhibited the growth of *Salmonella* Typhimurium, while a negative control from the *OmpA SS* strain had no effect on the *Salmonella* growth (Figure 4B). The scanning-electron micrographs also revealed that the cell-free supernatant from the *OmpA SS::MP-V1* strain effectively caused cellular lysis through the damage of the *Salmonella* membrane via pore formation, whereas a negative control from the *OmpA SS* strain did not (Figure 4C). Thus, these results clearly prove that the cell-free supernatant, produced by the *E. coli* system, contains the active MP-V1, forming pores into the *Salmonella* membrane. 

### 2.4. Anti-Salmonella Activity Modulation of the Cell-Free Supernatant Using Protease Inhibitors

**Figure 5:**
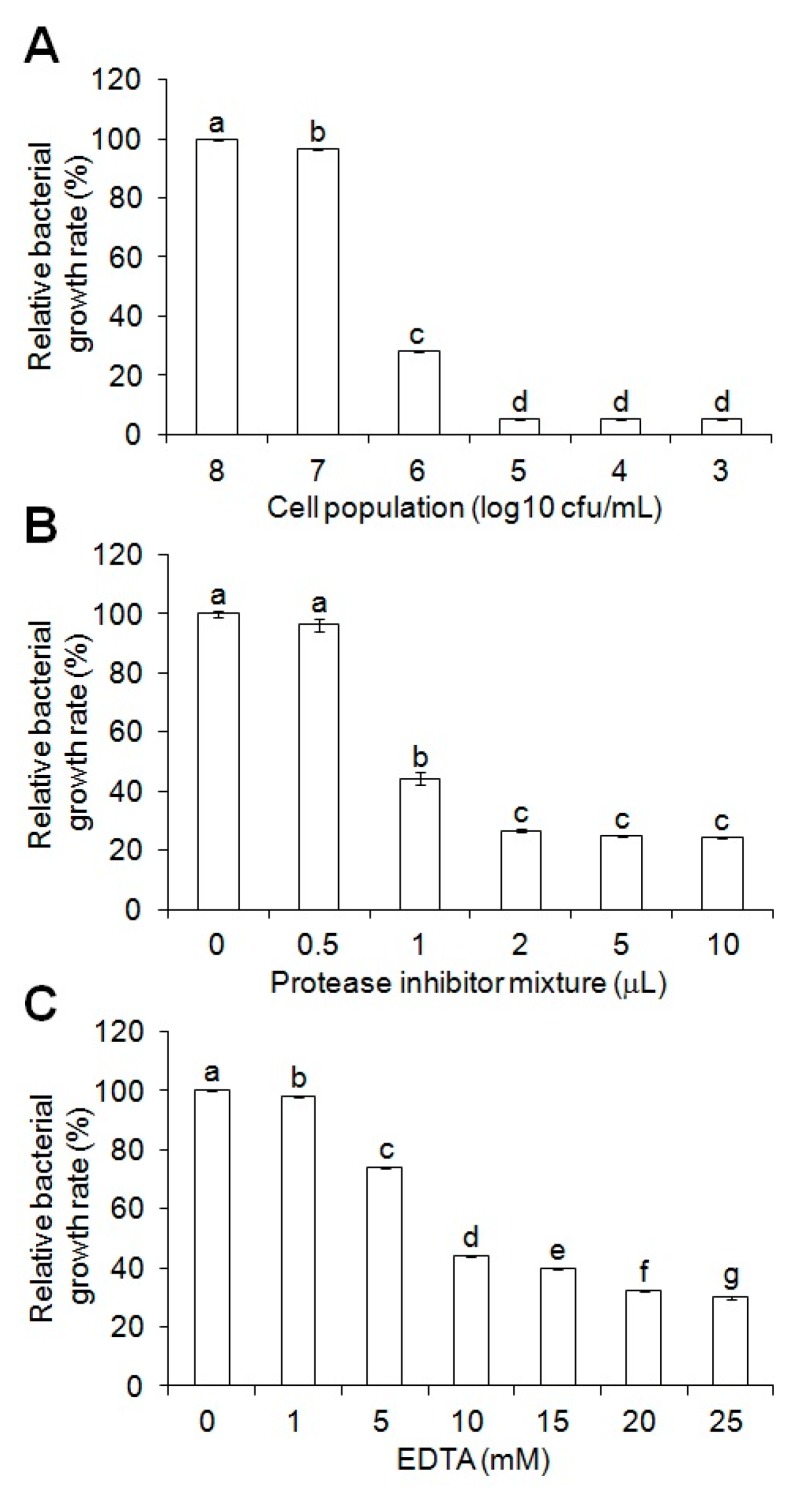
Effect of protease inhibitors on antimicrobial activities of the cell-free supernatant including MP-V1 in increased *Salmonella* population density. (**A**) Examination of antimicrobial activity of the cell-free supernatant including MP-V1 according to the *Salmonella* population density. The antimicrobial activity of 100 μL cell-free supernatant from the *OmpA SS::MP-V1* strain was examined against 10^3^ to 10^8^ cfu/mL of *Salmonella* Typhimurium. (**B**,**C**) The effect of the protease inhibitor cocktail and EDTA on antimicrobial activities of the cell-free supernatant including MP-V1 in increased *Salmonella* population density. The effect of a protease inhibitor cocktail (**B**) and EDTA (**C**) on antimicrobial activities of 100 μL cell-free supernatant from the *OmpA SS::MP-V1* strain was examined against 10^8^ cfu/mL of *Salmonella* Typhimurium using 0.5 to 10 μL doses and 1 to 25 mM doses, respectively. Data are means ± SE (*n* = 3). Different letters indicate significant differences by the one-way ANOVA/Duncan (*p* < 0.05).

When antimicrobial activity of 100 μL of the cell-free supernatant was examined against 10^3^ to 10^8^ cfu/mL populations of *Salmonella* Typhimurium, there were no antimicrobial activities against the 10^7^ and 10^8^ cfu/mL populations, while inhibitory effects on the bacterial growth rate against the 10^3^ to 10^6^ CFU/mL populations were observed (Figure 5A). Accordingly, as in Figure 2, the effect of the protease inhibitor cocktail on antimicrobial activities of 100 μL of the cell-free supernatant against 10^8^ cfu/mL of *Salmonella* Typhimurium was investigated to increase its antimicrobial activities against the increased population. As shown in Figure 5B, the protease inhibitor cocktail exhibited a dose-dependent effect on the increase of antimicrobial activity against the 10^8^ cfu/mL population. EDTA also effectively increased antimicrobial activity against the increased population of *Salmonella* Typhimurium (Figure 5C). Taken together, our results represent that the *E. coli* secretion system producing the active MP-V1 can be considered together with protease inhibitors as a successful strategy for its practical application.

## 3. Discussion

### 3.1. Protease Inhibitors Can Modulate the Anti-Salmonella Activity of MP-V1 through Avoidance of the Inoculum Effect

Antimicrobial agents often decrease in their activity with increasing density of the starting bacterial population, and this phenomenon is known as the inoculum effect (IE) [29]. We identified that MP-V1 showed no anti-*Salmonella* activities at its MICs, determined in 10^6^ cfu/mL of population, against the increased population (10^8^ cfu/mL) of three different *Salmonella* serotypes, suggesting that due to the IE, its MICs might increase significantly when the number of bacteria inoculated was increased to 10^8^ cfu/mL. The IE is known to be generally attributed to enzymatic degradation of the antimicrobial agents, despite the recent studies reporting other potential mechanisms, such as heat-shock-mediated growth instability, intercellular signaling between resistant and sensitive cells, and so on [29,30,31]. In addition, it has been reported that bacteria have an intrinsic AMP resistance mechanism through proteolysis using their proteases [21,22]. Thus, we investigated the effect of a protease inhibitor cocktail (Sigma-Aldrich, Milwaukee, WI, USA) on antimicrobial activities of MP-V1 at the MICs when the *Salmonella* inoculum density was increased to 10^8^ cfu/mL. The inhibitor cocktail showed a significantly dose-dependent effect on the increase of its antimicrobial activity against 10^8^ cfu/mL of all three serotypes (Figure 2), indicating that they contributed to lowering threshold levels of MP-V1 at the increased population density. Furthermore, when each of the inhibitors were examined, as in the inhibitor cocktail, all inhibitors used, except pepstatin A, showed significantly dose-dependent effects on the increase of antimicrobial activity against 10^8^ cfu/mL of all three serotypes (Figure 2). The inhibitor cocktail (Sigma-Aldrich, Milwaukee, WI, USA) used in this study is optimized commercially for only bacterial uses and is a mixture of inhibitors including 23 mM AEBSF, 2 mM bestatin, 0.3 mM pepstatin A, 0.3 mM E-64 and 100 mM EDTA, which inhibit serine proteases, aminopeptidases, aspartic acid proteases, cysteine peptidases and metalloproteases, respectively [32]. Accordingly, this indicates that the IE of MP-V1 against the increased *Salmonella* population density is at least partly caused by bacterial proteases, such as serine proteases, aminopeptidases, cysteine peptidases, or metalloproteases, except for aspartic acid proteases inhibited by pepstatin A. Therefore, these results suggest that protease inhibitors can be used as effective tools for modulating anti-*Salmonella* activity of toxins, such as MP-V1 in the increased bacterial population density, through avoidance of the IE. In addition, the anti-*Salmonella* activity modulation in the range of 10^8^ cfu/mL has a very important meaning for industrial applications, because that population density represents the general number of bacteria that grow in a culture medium.

### 3.2. Efficient Production of Active MP-V1 Using the OmpA SS-Mediated E. coli Secretion System

Obtaining efficiently potent AMPs, such as venom toxins, at low unit cost is a bottleneck in their practical application because their chemical synthesis is very expensive and the over-collection of crude venom extracts for their purification may result in ecosystem destruction [23,24]. Accordingly, instead of the above conventional methods, overexpression of an AMP in bacteria using recombinant technologies has been considered as an attractive strategy for its efficient production [24]. However, their bacterial toxicity also limits the use of the bacterial expression system for their efficient production and, thus, very few AMPs have been produced successfully in bacterial expression systems [24]. For example, AMPs such as moricin and cecropin from silkworms, defensin from humans and OG2 from frogs have been produced in bacterial expression systems using carrier proteins such as maltose-binding protein (MBP), glutathione *S*-transferase (GST), or thioredoxin (Trx) for the soluble expression of AMPs [33,34,35,36]. Furthermore, a recent *E. coli* expression system using green fluorescent protein (GFP) as a scaffold showed the efficient production of AMPs, such as protegrin-1 and PMAP-36 from pig, buforin-2 from toad and bactridin-1 from scorpion venom, in high yields [24]. However, the above bacterial expression systems still have a disadvantage in needing an additional step, such as chemical or enzymatic digestion of the MBP, the GST, the Trx or the GFP that is fused to AMPs, which may increase the production cost [24,33,34,35,36]. 

**Figure 6:**
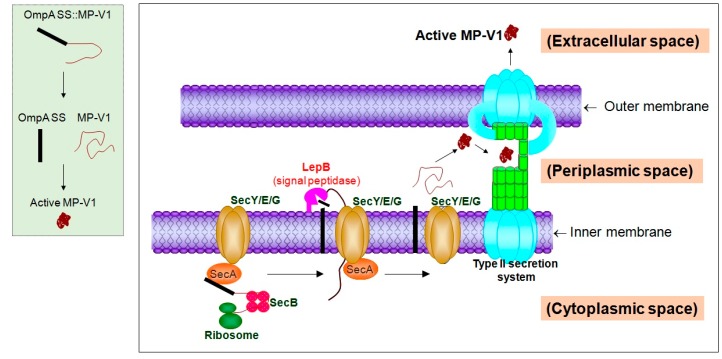
Secretion of MP-V1 to the extracellular space through the Sec-dependent type II secretion system. In the cytosol, the OmpA SS, a signal sequence of the *OmpA SS::MP-V1*, a translated target protein, is recognized by the SecA protein, which guides it to the plasma membrane, and the random folding of the target protein can be prevented by SecB, a chaperone protein. Subsequently, the OmpA SS fusion protein is translocated to the periplasm through the SecYEG complex in the inner membrane, the OmpA SS signal peptide is digested by the LepB, a peptidase, and then the digested MP-V1 is released freely into the periplasm. Finally, the digested peptide is secreted as the active MP-V1 to the extracellular space through the type II secretion system consisting of the general secretory proteins (GSPs). The left green box summarizes the procedure from the *OmpA SS::MP-V1* protein to the active MP-V1.

For efficient production of MP-V1, we constructed an *E. coli* secretion system expressing OmpA SS fusion peptides (Figure 4A). The OmpA SS was used as a signal peptide for delivery of the MP-V1 into the cell-free supernatant and in general, it exerts its role for the secretion of target peptides through the Sec-dependent type II secretion system consisting of Sec and GSPs (Figure 6). In detail, the SecA protein, existing in the cytosol, recognizes the OmpA SS, a signal sequence of a translated target protein, such as the *OmpA SS::MP-V1*, and then guides it to the plasma membrane (Figure 6) [37,38,39,40]. In the cytosol, random folding of the target protein can be prevented by SecB, a chaperone protein (Figure 6) [41]. Subsequently, the OmpA SS fusion protein is translocated to the periplasm through the SecYEG complex in the inner membrane [42], the OmpA SS signal peptide is digested by the LepB, a peptidase, and then the digested protein, such as MP-V1, is released freely into the periplasm (Figure 6) [43]. Finally, the digested protein is secreted to the extracellular space through the type II secretion system consisting of the GSPs (Figure 6) [26,27,28]. Through the *E. coli* secretion system, we obtained a cell-free supernatant from the *OmpA SS::MP-V1* strain, which dose-dependently inhibited the growth of *Salmonella* Typhimurium (Figure 4B) and formed pores into the *Salmonella* membrane (Figure 4C), while the one from the *OmpA SS* strain, a negative control, did not (Figure 4B,C). Thus, these results strongly support the hypothesis that the cell-free supernatant from the *OmpA SS::MP-V1* strain contains the active MP-V1, suggesting that our *E. coli* system can be a simple and efficient strategy, as opposed to previous methods that need an additional step, such as chemical or enzymatic digestion of the fusion tags such as the MBP, the GST, the Trx or the GFP.

## 4. Conclusions

We successfully modulated the antimicrobial activity of MP-V1 in an increased *Salmonella* population density by avoiding the IE through the use of protease inhibitors, and also showed that the *OmpA SS*-mediated *E. coli* secretion system is an efficient method to produce active MP-V1 in a cell-free supernatant and can be used together with protease inhibitors in an increased *Salmonella* population density (Figure 5). Altogether, these results suggest that our *E. coli* secretion system combined with protease inhibitors may be an attractive strategy for the practical application and production of AMPs such as venom toxins.

## 5. Materials and Methods

### 5.1. Materials

The three *Salmonella* serotypes, *Salmonella* Typhimurium, *Salmonella* Enteritidis and *Salmonella* Gallinarum (Table 1), were obtained from Dr. Jin Hur (Chonbuk National University, Iksan, Korea). The synthetic MP-V1 used in the previous study [25] was also used in this study. The protease inhibitor cocktail for bacterial use and the protease inhibitors, including AEBSF, bestatin, pepstatin A, E-64 and EDTA, were purchased from Sigma-Aldrich (Milwaukee, WI, USA). An *E. coli* strain and plasmids, used for the construction of the *E. coli* secretion system, are listed in Table 1, and Top10 (an *E. coli* competent cell) and the T-vector were purchased from Invitrogen (Carlsbad, CA, USA) and Promega (Madison, WI, USA), respectively.

### 5.2. Analysis of Minimal Inhibitory Concentration (MIC)

MIC assays of synthetic MP-V1 against *Salmonella* Typhimurium, *Salmonella* Enteritidis and *Salmonella* Gallinarum were performed by the microtiter plate method. The synthetic MP-V1 was dissolved to an appropriate concentration (10 mg/mL) and then applied for the assay of antimicrobial activity.

The precultured strains were used for the MIC assay via adjusting to 10^6^ cfu/mL or an appropriate concentration. Generally, the concentrations of synthetic MP-V1 applied for the MIC assay were 0, 25, 50, 100 and 250 μg/mL. After adding the reaction solution into a microtiter plate, the antimicrobial activity was observed for 16 h at 37 °C. The determination of MIC was performed by measurement at a wavelength of 600 nm (Multiscan GO, Thermo Scientific Co. Ltd., Rochester, NY, USA).

### 5.3. Examination of Antimicrobial Activity Depending on Protease Inhibitor

The starting stock solutions of each protease inhibitor used were prepared according to the information about the components of the protease inhibitor cocktail (Sigma-Aldrich, Milwaukee, WI, USA), comprising 23 mM AEBSF, 2 mM bestatin, 0.3 mM pepstatin A, 0.3 mM E-64 and 100 mM EDTA. The determination of MIC was performed at 10^8^ cfu/mL and each protease inhibitor concentration as indicated in Figure 2 and Figure 5B. The procedure for the determination of the antimicrobial activity was carried out in the same manner as described above.

### 5.4. Plasmid Construction for the Secretion of MP-V1 and Transformation into a General Host Strain

Nucleotide sequences of *OmpA SS* and *MP-V1* were collected via the National Center for Biotechnology Information (NCBI) and a previous study [25], respectively. The collected *OmpA SS* nucleotide sequence was designed to connect directly to the translational start site derived from the *Ptrc* promoter, and the *MP-V1* was also designed to directly connect *OmpA SS*. The *OmpA SS* was fused directly to the *Ptrc* promoter without *MP-V1*, and was designed to be used as a negative control. The designed artificial genes were prepared by an artificial-gene synthesis (Bioneer Corp., Daejon, Korea) and then cloned into T-vector (Promega, Madison, WI, USA), finally resulting in the pMMP319 and pMMP320 (Figure 4 and Table 1).

General DNA manipulations were conducted as described by Sambrook et al. [44]. Plasmids were introduced into Top10 (Invitrogen, Carlsbad, CA, USA), *E. coli* competent cells, by heat-shock with RbCl_2_ treatment. Nucleotide sequencing was conducted by using an ABI 3730XI automatic sequencer (Applied Biosystems, Foster City, CA, USA). The *E. coli* strain and plasmids used for this study are listed in the Table 1.

### 5.5. Antimicrobial Activity Analysis of Cell-Free Supernatant from the E. coli Secretion System

The pMMP319 containing the *OmpA SS* and the pMMP320 containing the *OmpA SS::MP-V1* were transformed into Top10 cells, which were aerobically precultured at 37 °C until optical density at a wavelength of 600 nm is 0.5. The cultured broth was centrifuged for 20 min at 3000 rpm, the supernatant was recovered, and then the solution was filtered by a 0.2 μm syringe filter. The determinations of MICs were performed for each cell population and protease inhibitor, as indicated in Figure 4 and Figure 6. The procedure for the determination of the antimicrobial activity was carried out in the same manner as described above.

### 5.6. Scanning-Electron Microscope (SEM) Analysis

*Salmonella* cells treated by the cell-free supernatants from the *E. coli* secretion system were fixed with a volume fraction of 2.5% glutaraldehyde (Sigma-Aldrich, Milwaukee, WI, USA) for 24 h at 4 °C. The samples were rinsed with sterile PBS buffer thrice, and then dehydrated with 30%, 50%, 70%, 80%, 90% and 100% (*v*/*v*) graded ethanol, successively, with 15 min incubation at each concentration. The samples were dried at room temperature and sprayed with a gold coating before the SEM observation.

### 5.7. Statistical Analysis

The one- or two-way analysis of variance (ANOVA) was followed by Duncan test using IBM SPSS software (IBM Corp., Armonk, NY, USA). Results are expressed as means ± standard errors (SEs) of at least three independent experiments. Different letters and asterisks indicate significant differences (*p* < 0.05).

## Figures and Tables

**Table 1:** Bacterial strains and plasmids used for this study.

Strains or Plasmids	Genotypes or Phenotypes	Sources
Bacterial strains		
*E. coli*		
Top10	F-*mcr*A Δ(*mrr*-*hsd*RMS-*mcr*BC) F80*lac*Z ΔM15 Δ*lac*X74 *rec*A1 *ara*Δ139 Δ(*ara-leu*)7697 *gal*U *gal*K *rps*L (Strr) *end*A1 *nup*G	Invitrogen
*Salmonella*		
HJL331	*Salmonella* Typhimurium HJL331, Wild type, Sm^R^ (isolated from swine)	Chonbok National University, Korea
HJL462	*Salmonella* Gallinarum HJL462, Wild type, Na^R^ (isolated from chicken)	Chonbok National University, Korea
HJL390	*Salmonella* Enteritidis HJL390, Wild type, Cm^R^ (isolated from swine)	Chonbok National University, Korea
**Plasmids**		
T-vector	Cloning vector; pUC*ori* Amp^R^	Promega
pMMP319	A T-vector derivative harboring *OmpA* *SS*	This study This study
pMMP320	A T-vector derivative harboring *OmpA* *SS::MP-V1*	
